# Pharmacological Property and Cytotoxic Effect Showing Antiproliferative Potency in Human Melanoma Cell Lines (A375) of *Combretum racemosum* P. Beauv. Leaf and Root Extracts Used in Benin

**DOI:** 10.3390/antiox13010031

**Published:** 2023-12-22

**Authors:** Durand Dah-Nouvlessounon, Michaelle Chokki, Ismaël M. S. Hoteyi, Fidèle Fassinou, Floricuta Ranga, Florinela Fetea, Zorita Diaconeasa, Dan Vodnar, Bianca Furdui, Farid Baba-Moussa, Rodica Mihaela Dinica, Ramona Suharoschi, Lamine Baba-Moussa

**Affiliations:** 1 Laboratory of Biology and Molecular Typing in Microbiology, Department of Biochemistry and Cell Biology, Faculty of Sciences and Techniques, University of Abomey-Calavi, Cotonou 05BP1604, Beninsmihot@yahoo.fr (I.M.S.H.); fassinoufidele@gmail.com (F.F.); 2 Department of Chemistry, Physics and Environment, “Dunarea de Jos” University of Galati, 47 Domneasca Street, 800008 Galati, Romania; bfurdui@ugal.ro; 3 Laboratoire de Microbiologie et de Technologie Alimentaire, FAST, Université d’Abomey-Calavi, 01BP: 526 ISBA-Champ de Foire, Cotonou 01BP526, Benin; michaellechokki@gmail.com (M.C.); fbmouss@yahoo.fr (F.B.-M.); 4 Faculty of Food Science and Technology, University of Agricultural Sciences and Veterinary Medicine Cluj-Napoca, 3-5 Calea Manastur Street, 400372 Cluj-Napoca, Romaniadan.vodnar@usamvcluj.ro (D.V.); ramona.suharoschi@usamvcluj.ro (R.S.)

**Keywords:** polyphenol compound, biological activities, MTT cytotoxic test, plant extract

## Abstract

*Combretum racemosum*, a plant from the Combretaceae family, is traditionally used in Benin for various health problems. However, scientific research on Beninese samples of this plant is limited. The aim of this study was to identify and assess the bioactive compounds in the plant’s leaves and roots. Initial screening involved analyzing powders derived from these parts for total polyphenols, flavonoids, and both condensed and hydrolyzable tannins. The polyphenolic compounds were analyzed using HPLC-DAD-ESI-MS. To evaluate the plant’s antimicrobial properties, the agar diffusion method was employed, while FRAP and DPPH assays were used to determine its antioxidant capacity. For anti-inflammatory activity, the study utilized tests for in vitro protein denaturation inhibition and in vivo acute edema induced by carrageenan. Additionally, an antiproliferative assay was conducted using the human melanoma cell line A375. The analysis revealed the presence of significant polyphenolic compounds in both the leaf and root extracts of *C. racemosum*. Notably, compounds like Pedunculagin, Vescalagin, Casuarictin, and Digalloyl-glucoside were abundant in the leaves, with Vescalagin being especially predominant in the roots. The study also found that the dichloromethane extracts from the leaves and roots exhibited bactericidal effects on a substantial percentage of meat-isolated strains. Moreover, the antioxidant activities of these extracts were confirmed through FRAP and DPPH methods. Interestingly, the dichloromethane root extract showed strong activity in inhibiting thermal albumin denaturation, while the water–ethanol leaf extract demonstrated significant edema inhibition. Finally, the study observed that *C. racemosum* extracts reduced cell viability in a dose-dependent manner, with leaf extracts showing more pronounced antiproliferative effects than root extracts. These findings highlight the potential of *C. racemosum* leaves and roots as sources of compounds with diverse and significant biological activities. In conclusion, *C. racemosum*’s leaves and roots exhibit promising biological activities, highlighting their potential medicinal value.

## 1. Introduction

Since the earliest stages of human evolution, plants have been a fundamental part of our existence, fulfilling essential needs beyond just nourishment. Humans have long tapped into the medicinal properties of plants, recognizing them as sources of natural molecules with diverse biological activities. In the modern era, with advancements in science, we have developed various methods to synthesize molecules for extensive applications in healthcare, particularly for treating communicable and metabolic diseases.

However, the overuse and mismanagement of synthetic drugs in treating infectious diseases have led to a significant rise in antimicrobial resistance, a grave health concern. In 2019, it was estimated that out of 13.7 million infection-related deaths, nearly 7.7 million were linked to 33 specific bacterial pathogens, including *Staphylococcus aureus*, *Escherichia coli*, *Streptococcus pneumoniae*, *Klebsiella pneumoniae*, and *Pseudomonas aeruginosa* [1]. Alarmingly, almost 4.95 million of these deaths were attributed to antimicrobial resistance, with bacterial resistance accounting for 1.27 million cases [2].

This growing antimicrobial resistance exacerbates infections and complicates treatment. In response to infection, the body produces various mediators such as inflammatory cytokines and chemokines. The intricate role of these mediators often triggers complex biological responses in patients [3,4]. Furthermore, certain cytokines/chemokines like TNF-α, IL-18, and RANTES are believed to contribute directly to tumor growth by stimulating receptors on tumor cells. This intricate interplay of microbial resistance and the body’s response underscores the need for continued research and development in medical science to address these emerging health challenges [5].

The presence of antioxidants, which counteract radicals, plays a pivotal role in controlling inflammation. These antioxidants are capable of reducing or completely preventing inflammatory responses. This is due to the fact that free radicals activate certain transcription factors and pro-inflammatory genes, initiating inflammation. This, in turn, prompts immune cells to produce a range of cytokines and chemokines, drawing more immune cells to the sites of oxidative stress or infection. This escalation in immune cell activity leads to the further production of free radicals, causing oxidative stress and subsequent tissue damage [6]. For instance, TNF-α is known to increase the production of reactive oxygen intermediates (ROIs) by neutrophils and other cells. Additionally, interleukin-1-β (IL-1-β), TNF-α, and interferon (IFN)-ϒ drive the production of inducible nitric oxide synthase in inflammatory and epithelial cells [7]. This cycle of inflammation and oxidative stress can eventually damage healthy stromal and neighboring epithelial cells, possibly leading to carcinogenesis over time [8].

In the realm of carcinomas, malignant melanoma is notably the most aggressive form of skin cancer, known for its rapid spread and high invasiveness [9]. Presently, the development of effective systemic treatments for this cancer is challenging, primarily due to its resistance to many drugs [10].

In recent times, the limitations of synthetic products have become evident, especially in managing pathological challenges. The need for alternative solutions is pressing, particularly in the development and use of antimicrobial, non-steroidal anti-inflammatory, antioxidant, and anticancer agents. These are crucial in reducing the occurrence of complications, adverse effects, and relapses associated with conventional treatments [11]. A promising approach lies in harnessing the power of medicinal plants, tapping into traditional knowledge systems for effective remedies.

One such plant is *Combretum racemosum*, belonging to the Combretaceae family and commonly found in Sub-Saharan Africa. This medicinal plant has been the focus of various studies, revealing its rich array of bioactive compounds [12,13,14,15,16]. Species within the Combretum genus are particularly noted for their significant anticancer properties [17,18]. Additionally, *C. racemosum* has been recognized for its diverse biological activities. It has shown effectiveness as an anthelmintic [18] and in combating trypanosomal and antiplasmodial infections [19,20]. Its anti-inflammatory and antioxidant properties are also well-documented [13,21,22], along with its potential in treating urinary and gastrointestinal infections [15]. These findings underscore the importance of integrating medicinal plants like *C. racemosum* into modern therapeutic strategies.

In Benin, the local communities widely use *Combretum racemosum* (*C. racemosum*) for treating various diseases, yet there is scant scientific research on the samples gathered from this region [23]. This study aims to bridge this knowledge gap by identifying phenolic compounds in Benin-sourced *C. racemosum* samples using LC-MS (liquid chromatography–mass spectrometry). The research will not only focus on pinpointing these compounds but also evaluate the plant’s antimicrobial and antioxidant capabilities, as well as its anti-inflammatory properties. A significant aspect of the study is to investigate the effects of *C. racemosum* on human melanoma cells, thereby assessing its potential as a preliminary anticancer agent. This research could provide valuable insights into the therapeutic properties of *C. racemosum*, particularly as used in traditional Beninese medicine.

## 2. Materials and Methods

### 2.1. Plant Material and Extract Preparation

Samples of *C. racemosum*, including both leaves and roots, were gathered from the Dangbo district in southern Benin, specifically between the coordinates N06°35′19.7″ and E002°33′15.9″. The samples were collected from species growing naturally near village housing huts and without any chemical treatment. These specimens were then deposited for official certification at the National Herbarium of Benin, located at the University of Abomey-Calavi in Cotonou, under the supervision of Herbarium conservator Prof. Hounnankpon Yedomonhan. The certification was recorded under voucher No. YH758/HNB.

In preparation for analysis, the leaf samples were dried at a consistent temperature of 23 ± 2 °C for a duration of 14 days, while the root samples underwent the same drying process for 28 days. Post-drying, these samples were finely ground using a Retsch grinder (model SM 2000/1430/Upm/Smf, Haan, Germany) to ensure uniformity for subsequent extraction.

For the extraction process, 1 g of each powdered sample was treated with 100 mL of an appropriate solvent. This extraction was facilitated by ultrasonication at a frequency of 35 Hz for 2 h, employing a variety of six solvents to optimize extraction efficiency. These solvents included water, a water–ethanol mixture with a volume proportion of 30:70, ethanol, methanol, dichloromethane, and acetone. The resulting solutions were then filtered and concentrated using a Heidolph Laborota 400 Rotovap rotary evaporator (Schwabach, Germany) to obtain the final extracts for analysis.

### 2.2. Preliminary Phytochemical Screening

The methodology for identifying bioactive compounds (secondary metabolites) in the powder of *C. racemosum* leaves and roots was based on the protocol established by Hounghton and Raman [24]. This approach was specifically designed to detect a range of secondary metabolites, including those from key groups such as nitrogenous compounds, polyphenolic and terpenic compounds, and glycosides. By employing this protocol, the study aimed to comprehensively explore the diverse bioactive constituents present in *C. racemosum*, which could contribute to its medicinal properties.

### 2.3. C. racemosum Total Polyphenol Content Determination

The measurement of total polyphenol content in the extracts of *C. racemosum* leaves and roots was conducted in accordance with the method described by Dicko et al. [25], utilizing the Folin–Ciocalteu reagent. For this assay, 25 microliters (25 µL) of Folin–Ciocalteu’s reagent, diluted to 50% *v*/*v*, was combined with 10 µL of the plant extracts, concentrated to 1 mg/mL *w*/*v*. This mixture was then allowed to incubate for 5 min at room temperature. Following this, 25 µL of 20% *w*/*v* sodium carbonate (Na_2_CO_3_) and ultrapure water were added, bringing the total volume in each well to 200 µL. To control for any substances that might interfere with the assay, blanks were prepared by substituting the reagent with ultrapure water.

After a further 30 min incubation period, the absorbance of the mixture was measured at a wavelength of 760 nm using a Tecan Pro 200 multiwell plate reader, manufactured by Tecan Trading AG, Männedorf, Switzerland. To ensure the reliability and consistency of the results, each assay was performed a minimum of three times. The standard used for comparison in these tests was gallic acid, ranging from 0 to 500 µg/mL. The findings were then expressed as equivalents of gallic acid per 100 g of the extract, providing a standardized metric for assessing the polyphenolic content in these plant extracts.

### 2.4. C. racemosum Total Flavonoid Content Determination

The total flavonoid content in each sample was determined following the aluminum trichloride (AlCl_3_) colorimetric method, as outlined by Culdalbeanu et al. [26]. In this procedure, each well of a 96-well microplate received a mixture of 100 µL of 2% AlCl_3_ and 100 µL of the specific extract solution from *C. racemosum* leaves and roots. After a 15 min incubation period, the absorbance of these solutions was measured at 415 nm. This measurement was performed using a Tecan Pro 200 multiwell plate reader (Tecan Trading AG, Männedorf, Switzerland).

The absorbance of each sample was then compared against a blank, which consisted of a combination of 100 µL of the extract solution and 100 µL of methanol. Additionally, the results were calibrated against a Quercetin standard curve, which ranged from 0 to 50 µg/mL and had an R2 value of 0.99, ensuring high accuracy and reliability of the measurements. The flavonoid content in the extracts was subsequently expressed in terms of micrograms of Quercetin equivalents per 100 g of extract, providing a standardized quantification of the flavonoid levels present in the *C. racemosum* samples.

### 2.5. C. racemosum Condensed Tannin Content Determination

The quantification of condensed tannin content in the extracts followed the procedure established by Belem-Kabré et al. [27]. This method involved mixing 1 mL of the extract, prepared at a concentration of 5 mg/mL, with 2 mL of 1% vanillin solution in 70% sulfuric acid. This mixture was then incubated at 20 °C for 15 min in a water bath. Following incubation, the absorbance of the mixture was measured at 500 nm using the Biomate™ 3 Series Spectrophotometers (Thermo Scientific, Dreieich, Germany). The content of condensed tannins in the samples was calculated using a specific formula provided in the method. This approach allows for precise measurement of the condensed tannin concentration in the extracts, contributing valuable data to the study, using the formula:

$$C\left(mgEC\right)=5.2\times 10^{-2}\times \frac{\left(A\times V\right)}{P}$$

where 5.2 × 10^−2^ = constant in equivalents of cyanidin, A = absorbance, V = volume of extract, and P = weight of extract. The results were expressed as milligrams of cyanidin equivalents (CE) per gram of extract.

### 2.6. C. racemosum Hydrolyzable Tannin Content Determination

To measure the hydrolyzable tannin content in *C. racemosum* leaf and root extracts, we applied the method previously described by the same authors [27]. In this procedure, 1 mL of each extract (both leaves and roots) was combined with 3.5 mL of a ferric chloride solution, which is a concentration of 10^−2^ M FeCl_3_ in HCl. This mixture was then incubated for a brief duration of 30 s. After incubation, the absorbance of each sample was measured at a wavelength of 660 nm using the Biomate™ 3 Series Spectrophotometers, manufactured by Thermo Scientific in Bremen, Germany. The content of hydrolyzable tannins in the extracts was subsequently calculated using a specific formula, allowing for the accurate determination of their concentration in the samples. This quantitative analysis is crucial for understanding the potential biological and therapeutic properties of the *C. racemosum* extracts:

$$C(mgEGA)=\frac{\left(A\times PM\times V\times FD\right)}{Ɛ\;mole\times P}$$

where A = absorbance, PM = weight of gallic acid (170.12 g/mol), V = volume of extract, FD = dilution factor, ε mole = 2169 (constant in equivalents of gallic acid), and P = extract weight.

### 2.7. Polyphenol Analysis by HPLC-DAD-ESI-MS

For the analysis of *C. racemosum* leaf and root extracts, a sophisticated liquid chromatography setup was employed. This system included an HP-1200-LC equipped with a quaternary pump, an autosampler, a Diode Array Detector (DAD), and an MS-6110 single quadrupole API-electrospray detector, all provided by Agilent Technologies, Santa Clara, CA, USA. The detection of phenolic compounds was enhanced by varying the fragmentor voltage in the range of 50–100 V in ESI+ mode.

The chromatographic analysis utilized a Kinetex XB-C18 column (5 μm; 4.5 × 150 mm i.d.) sourced from Phenomenex, USA. The mobile phase for this analysis comprised two solvents: water acidified with 0.1% acetic acid (solvent A) and acetonitrile, also acidified with 0.1% acetic acid (solvent B). A specific multistep linear gradient was applied for elution over a 30 min period, with an elution flow rate set at 0.5 mL/min and a column temperature maintained at 25 ± 0.5 °C. The gradient program began with 5% solvent B for 2 min, then a gradual increase to 90% solvent B over 20 min, maintained at 90% for 4 min, followed by a return to 5% B over the final 6 min.

**Figure 1:**
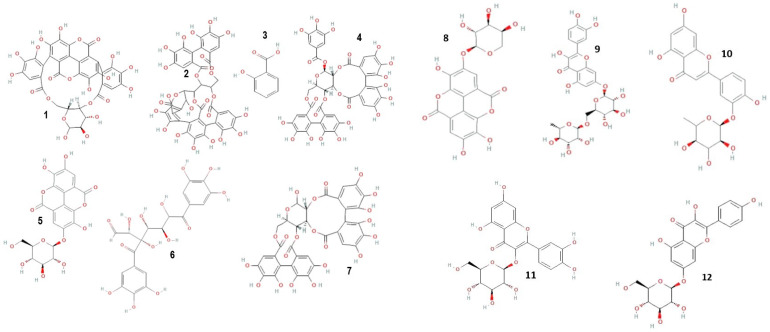
Structure of some phenolic and flavonoid compounds identified in *C. racemosum* leaf and root extracts. **1**: Punicalin; **2**: Vescalagin; **3**: 2-Hydroxybenzoic acid; **4**: Casuarictin; **5**: Ellagic acid-glucoside; **6**: Digalloyl-glucoside; **7**: Pedunculagin; **8**: Ellagic acid-arabinoside; **9**: Quercetin-rutinoside; **10**: Luteolin-rhamnoside; **11**: Quercetin-glucoside; **12**: Kaempferol-glucoside.

Mass spectrometric detection focused on positively charged ions, using the Scan mode for enhanced sensitivity. The experimental conditions for this phase included a gas temperature of 350 °C, nitrogen flow at 7 L/min, nebulizer pressure at 35 psi, capillary voltage at 3000 V, a fragmentor set at 100 V, and a mass-to-charge ratio (*m*/*z*) range of 120–1500. Chromatograms were recorded at wavelengths of λ = 280 and 340 nm, with data acquisition managed using the Agilent ChemStation B.04.01 LC 1200 series software. This comprehensive analytical approach ensured precise identification and quantification of the phenolic compounds in the *C. racemosum* extracts. Figure 1 shows the structure of some identified compounds in *C. racemosum* leaf and root extracts. For the identification of phenolic compounds, we utilized a public database, the Phenol-Explorer database, as a reference tool (http://phenol-explorer.eu access on 15 November 2022).

### 2.8. Antimicrobial Activity of C. racemosum Leaf and Root Extracts

#### 2.8.1. Tested Microorganisms

In this study, five reference strains were utilized: *Staphylococcus aureus* ATCC 6538P, *Listeria monocytogenes* ATCC 19114, *Escherichia coli* ATCC 25922, *Salmonella enteritidis* ATCC 13076, and *Candida albicans* ATCC 10231. Additionally, seven meat-isolated *Staphylococcus* strains were included. These meat-isolated strains, part of the collection at the Laboratory of Biology and Molecular Typing in Microbiology at the University of Abomey-Calavi, Benin, were originally isolated from pork in a study by Attein et al. [28]. All strains, both reference and meat-isolated, were revived and prepared for analysis as detailed in our previous work [29], ensuring their viability and consistency for the tests conducted in this study.

#### 2.8.2. Antibiogram

The disc diffusion method [30] was employed to assess the sensitivity of meat-isolated Staphylococcus strains to extracts from *C. racemosum* leaves and roots. In this procedure, Mueller–Hinton Agar Petri dishes were first inoculated with 1 mL of an appropriately adjusted inoculum, set at a density of 1.5 × 10^8^ CFU/mL. Subsequently, under sterile conditions, five wells were created on the surface of the inoculated Mueller–Hinton agar in each dish.

Into these wells, 25 µL of the *C. racemosum* leaf and root extract solutions, each at a concentration of 20 mg/mL, were carefully placed aseptically. The Petri dishes were then incubated at 37 °C for a duration of 24 h. Following the incubation period, the dishes were inspected for the formation of inhibitory zones around the wells, which would indicate the antimicrobial effectiveness of the extracts. To ensure the validity and consistency of the results, this entire process was repeated in triplicate for each sample. This method allowed for a thorough evaluation of the antimicrobial potential of the *C. racemosum* extracts against the specified bacterial strains.

#### 2.8.3. Determination of the Minimum Inhibitory Concentration (MIC)

To determine the Minimum Inhibitory Concentration (MIC) of *C. racemosum* leaf and root extracts, the microdilution method was utilized, incorporating resazurin as a cell viability indicator. This method involved testing a range of extract concentrations, from 20 to 0.039 mg/mL, against the microbial strains.

In the process, 100 µL of nutrient broth (for bacterial strains) and TSB (trypticase soy broth) (for yeast) were dispensed into all wells of the microplate, from well 2 to 9. Subsequently, 100 µL of each extract was added into wells 1 and 2. This was followed by successive half dilutions from well 2 to well 9. Each well then received 100 µL of the corresponding inoculum, adjusted to 1.5 × 10^4^ CFU/mL, before the plates were incubated for approximately 24 h at 37 °C for bacterial strains and 30 °C for *Candida albicans*.

Post-incubation, 20 µL of resazurin was added to each well, and the plates were incubated again under the same conditions (37 °C for bacterial strains and 30 °C for *Candida albicans*) for an additional 2 h. The MIC was determined as the lowest extract concentration at which the color of the resazurin did not change from blue to pink, indicating the inhibition of microbial growth at that concentration. This method provided a precise and reliable measure of the antibacterial and antifungal efficacy of the *C. racemosum* extracts.

#### 2.8.4. Determination of the Minimum Bactericidal or Fungicidal Concentration (MBC or MFC)

The determination of the Minimum Bactericidal Concentration (MBC) for the bacteria and the Minimum Fungicidal Concentration (MFC) for the yeast in the study was conducted by subculturing. This process involved transferring the test dilutions from the MIC assay onto fresh Mueller–Hinton agar plates for bacterial strains and YPD agar plates for the yeast. These plates were then incubated under the same conditions as the MIC assay for an additional 18–24 h.

The MBC for bacteria and the MFC for yeast were identified as the lowest concentration of the extracts at which no visible growth of bacteria or yeast was observed [31]. This concentration is indicative of the ability of the extracts to not just inhibit, but to effectively kill the microorganisms. This critical step in the study provides an understanding of the bactericidal and fungicidal strengths of the *C. racemosum* extracts, essential for evaluating their potential therapeutic applications.

### 2.9. Antioxidant Activity of C. racemosum Leaf and Root Extracts

#### 2.9.1. 2,2-Diphenyl-1-picrylhydrazyl Assay

The DPPH-radical-scavenging activity of *C. racemosum* leaf and root extracts was evaluated using a method described by Chokki et al. [32]. For this assay, 100 µL of a 50 μM DPPH solution and 100 µL of the plant extracts, at a concentration of 200 μg/mL, were combined in each well of the microplate. The mixture was then left in the dark for 20–30 min at room temperature to allow for the reaction to occur.

Following this incubation period, the absorbance of the mixture was measured at 517 nm using a Tecan Pro 200 microplate reader, manufactured by Tecan Trading AG, Männedorf, Switzerland. A blank was simultaneously prepared under the same conditions, substituting the plant extract with 100 µL of the solvent used for dilution. The percentage of DPPH-radical-scavenging activity for the *C. racemosum* leaf and root extracts was then calculated using a formula previously established by Schmeda-Hirschmann et al. [33]:

$$Inhibitory\;Percentage\left(\%\right)=\frac{{Blank}^{’}s\;absorbance-{Sample}^{’}s\;absorbance}{{Blank}^{’}s\;absorbance}\times 100$$

This methodology provided a quantitative assessment of the antioxidant capacity of the extracts, an important aspect in evaluating their potential therapeutic benefits.

#### 2.9.2. Ferric-Reducing Antioxidant Power (FRAP) Assay

The FRAP assay for *C. racemosum* leaf and root extracts was performed following a method adapted to microplates, as described by Rumpf et al. [34]. Fresh FRAP reagent was prepared by combining 100 mL of acetate buffer (300 mM, pH 3.6) with 10 mL of a TPTZ (2,4,6-tripyridyl-s-triazine) solution (10 mM in 40 mM HCl) and 10 mL of FeCl_3_ (20 mM in ultrapure water). This reagent was then incubated at 37 °C for 10 min before use.

For the assay, 100 µL of methanol was first added to each well of a 96-well microplate. Starting from the first well, a half dilution series was created along the row with 100 μL of each sample. These dilutions were then mixed with 100 µL of the FRAP solution and incubated at 37 °C for 10 min. Post-incubation, the absorbances were measured at 593 nm using a Tecan Pro 200 microplate reader (Tecan Trading AG, Männedorf, Switzerland).

A calibration curve was established using Trolox (0.41–2.54 mM/mL) to quantify the antioxidant activity. The percentage of ferric-reducing power, indicating the antioxidant capacity of the extracts, was calculated using a formula as specified in the method. This assay provided a quantitative measure of the extracts’ ability to reduce ferric ions, a key indicator of their antioxidant potential.

$$\%Inh=\frac{Abs\;sample-Abs\;blank}{Abs\;sample}\times 100$$

The determination of the IC_50_ value, which represents the concentration required to achieve 50% inhibition, was carried out using a regression equation. This equation was derived from a curve that illustrated the relationship between the percentage of inhibition and the concentration of the *C. racemosum* plant extracts, as outlined in a previous study [35].

Additionally, the Trolox Equivalent Antioxidant Capacity (TEAC) value was calculated by considering the molar mass of Trolox and the concentrations of the extracts. This approach allows for a direct comparison of the antioxidant capacity of the plant extracts to that of Trolox, a well-known standard in antioxidant assays. By utilizing this methodology, the study provided a detailed and quantifiable assessment of the antioxidant potential of *C. racemosum* extracts, offering valuable insights into their efficacy as natural antioxidants.

$$C\left(mMET\right)=\left(\frac{Abs\;sample-bcal}{mcal}\right)\times \left(\frac{1}{MTrolox\times Cext}\right)$$

where Abs = absorbance; bcal = y-intercept of calibration curve; mcal = slope of the calibration curve; MTrolox = molar mass of Trolox; Cext = concentration of extract.

### 2.10. Protein Denaturation Inhibition Capacity of C. racemosum Leaf and Root Extracts

The in vitro anti-inflammatory activity of *C. racemosum* leaf and root extracts was assessed using a method previously described by Kabré et al. [36], which involves the use of egg albumin. In this assay, conducted in a 96-well microplate, 100 µL of the extracts (with concentrations ranging from 7.81 to 2000 µg/mL) were combined with 10 µL of egg albumin and 140 µL of phosphate-buffered saline (PBS, pH 6.4). This mixture was first incubated at 37 °C for 15 min, followed by heating at 70 °C for 5 min.

A blank sample was prepared under identical conditions, substituting the plant extracts with ultrapure water, to control for any changes unrelated to the extracts’ activity. The absorbance of each sample was then measured at 660 nm using a Tecan Pro 200 microplate reader (Tecan Trading AG, Männedorf, Switzerland). Diclofenac was used as a standard control in the experiment. The percentage of inhibition (% *Inh*) of albumin denaturation, indicative of the anti-inflammatory activity of the extracts, was calculated using the following formula:

$$\%\;Inh=\frac{Abs\;C-Abs\;S}{Abs\;C}$$

AbsC = absorbance of control; AbsS = sample absorbance.

This procedure provided a comprehensive evaluation of the anti-inflammatory potential of the *C. racemosum* extracts.

The IC_50_ value, representing the concentration necessary to achieve 50% inhibition of albumin’s thermal denaturation, was calculated using an equation obtained from a logarithmic curve. This curve was the result of a non-linear regression analysis, where the inhibition percentage was plotted against the varying concentrations of the plant extracts. This method provided a precise and quantifiable means of determining the efficacy of the *C. racemosum* extracts in inhibiting protein denaturation, a key indicator of their anti-inflammatory potential.

### 2.11. Carrageenan-Induced Paw Edema

#### 2.11.1. Animal and Ethical Approval

Wistar rats, specifically of the Exempt from Specific Pathogenic Organisms (EOPS) health status, were employed in this study. These rats were approximately eight weeks old and had body weights ranging between 150 g and 200 g. The research protocol for this study received approval from the Scientific Ethics Committee of the Doctoral School of Life Sciences at the Faculty of Science and Technology (FAST), University of Abomey Calavi (UAC), Benin. The approval was granted under the reference number UAC/FAST/EDSV/16945713, ensuring compliance with ethical standards and regulations for animal research.

#### 2.11.2. Experimental Design

Following a one-week acclimatization period at a stable temperature of 22 ± 2 °C and a 12/12 h light/dark cycle, the rats were randomly assigned into six groups, each comprising three animals. The anti-inflammatory activity of *C. racemosum* leaf and root extracts was evaluated using a method adapted from Khanna et al. [37]. The groups were organized as follows: Group 1 (negative control): received water; Group 2 (positive control): received diclofenac at a dosage of 50 mg/kg body weight; Group 3: administered 200 mg/kg orally of the leaf ethanol extract; Group 4: administered 200 mg/kg orally of the water–ethanol leaf extract; Group 5: administered 200 mg/kg orally of the root ethanol extract; and Group 6: administered 200 mg/kg orally of the root water–ethanol extract.

An hour before administering these treatments, the diameter (Do) of the right hind leg of each rat was measured using a digital caliper. Then, one hour post-oral treatment, 100 µL of a fresh 1% carrageenan solution was injected under the plantar pad of the right hind paw of each rat. The edema size was measured at hourly intervals for 5 h. From these measurements, the percentages of edema increase and inhibition were calculated using a specified formula, allowing for a detailed assessment of the anti-inflammatory effects of the *C. racemosum* extracts.

$$EI\left(\%\right)=\frac{Dt-Do}{Dt}\times 100$$

where EI (%) = edema increase percentage; Dt = average diameter of the right hind leg at time t; Do = average diameter of the right hind leg at time 0 (before treatment).

The edema increase inhibition (EII) rate (%) was calculated as follow:

$$EII\left(\%\right)=\frac{\left[EI\right]control\;group-[EI]treat\;group}{[EI]control\;group}\times 100$$

#### 2.11.3. Cell Proliferation Assay

The human melanoma cell line A375 was procured from the American Type Culture Collection (ATCC, Manassas, VA, USA). These cells were cultured as a monolayer at 37 °C in a humidified atmosphere of 5% CO_2_ and 95% air. The growth medium used was DMEM enriched with 10% fetal bovine serum, streptomycin (50 units/mL), and penicillin (100 units/mL).

For the experiment, 8 × 10^3^ A375 human melanoma cells per well were seeded in 96-well plates and allowed to adhere for 24 h. Subsequently, the medium in each well was replaced with fresh DMEM containing varying concentrations (0 to 50 μg/mL) of *C. racemosum* leaf and root ethanol extracts for an additional 24 h. Following this incubation period, the medium was replaced with MTT reagent (0.5 mg/mL) for 2 h. After discarding the MTT reagent, the formazan crystals formed were dissolved in DMSO.

The absorbance of the solubilized formazan, which is indicative of cell viability, was then measured at dual wavelengths of 550 nm (for the sample) and 630 nm (for background) using an HT BioTek Synergy microplate reader (BioTek Instruments, Winooski, VT, USA). This procedure allowed for the assessment of the cytotoxic effects of the *C. racemosum* extracts on the A375 melanoma cells.

#### 2.11.4. Statistical Analysis

The experimental data were systematically organized and analyzed using the Excel 2016 database. For graphical representation and further statistical analysis, GraphPad Prism^®^ software (version 8.0.2) was employed. Statistical analysis was conducted using ANOVA (Analysis of Variance) for multivariate analysis, complemented by Tukey’s post-hoc test to determine significant differences between groups. In this context, *p*-values less than 0.05 were deemed to indicate statistical significance. The outcomes of these analyses are presented in the format of mean ± standard deviation, ensuring a clear and precise representation of the data and their variability. This comprehensive approach to data analysis and presentation allows for a thorough and accurate interpretation of the experimental results.

## 3. Results

### 3.1. Phytochemical Screening

The outcomes of the phytochemical screening conducted on the powders derived from *C. racemosum* leaves and roots are presented in Table 1. The analysis revealed the presence of eleven (11) distinct subclasses of compounds within this species. These include tannins, catechic tannins, gallic tannins, flavonoids, anthocyanins, and saponosides in the roots, as well as alkaloids, quinone derivatives, mucilages, reducing compounds, and free anthracenics. However, the screening also identified certain compounds that were absent. Specifically, the leaves did not contain leuco-anthocyanins or saponosides, and there was a notable absence of cyanogenic derivatives, O-glycosides, and O-glycosides with reduced genins in both leaves and roots. This comprehensive phytochemical profile provides valuable insights into the chemical composition of *C. racemosum* and its potential therapeutic properties.

### 3.2. Total Polyphenol, Flavonoid, and Tannin Contents of C. racemosum Extracts

The secondary metabolite composition of *C. racemosum* extracts, highlighting the variability in polyphenol content across different extracts and plant parts, are presented in Table 2. For the leaves, the ethanol extract exhibited the highest polyphenol concentration at 5316.98 ± 99.15 mg GAE (gallic acid equivalents)/100 g. This was followed closely by the aqueous extract with 4953.62 ± 918.10 mg GAE/100 g, while the dichloromethane extract contained the least at 413.20 ± 12.56 mg GAE/100 g.

In the case of the roots, the ethanol extract again demonstrated the highest polyphenol content (5347.19 ± 99.76 mg GAE/100 g), but this time it was followed by the acetone extract, which had a content of 4654.54 ± 27.74 mg GAE/100 g. Notably, the aqueous extract of the roots also showed a significant polyphenol concentration of 4483.90 ± 975.23 mg GAE/100 g, which was comparable to that of the acetone extract. These findings provide a comprehensive overview of the polyphenolic profile of *C. racemosum*, indicating its potential richness in beneficial compounds.

Similar to polyphenols, the content of flavonoids in *C. racemosum* varied across different extracts and plant parts. Focusing on the leaves, the ethanol extract displayed a notably high flavonoid content of 13,157.10 ± 28.43 mg QE (Quercetin equivalents)/100 g. This concentration was remarkably 17-fold greater than that found in the acetone extract, which had the lowest flavonoid content at 756.68 ± 76.41 mg QE/100 g.

In the case of the roots, the ethanol extract again stood out with the highest flavonoid content, recorded at 10,888.12 ± 375.10 mg QE/100 g. By contrast, the methanol extract of the roots presented the lowest flavonoid content, measuring 920.44 ± 64.50 mg QE/100 g. These observations highlight the significant variation in flavonoid levels in different extracts of *C. racemosum*, underscoring the importance of extract selection based on desired flavonoid content for potential applications.

The lowest quantities among the compounds analyzed were found in the tannins. This observation indicates that the extracts are less rich in tannins (both condensed and hydrolyzable). Specifically, the content of condensed tannins in the leaves ranged from 25.48 ± 0.56 mg CE (Catechin equivalents)/g in the water–ethanol extract to 39.95 ± 1.42 mg CE/g in the methanol extract. In the roots, these levels varied from 22.92 ± 3.66 mg CE/g in the water–ethanol extract to 396.75 ± 12.76 mg CE/g in the dichloromethane extract. As for the hydrolyzable tannins, the dichloromethane extract of the leaves showed the lowest concentration at 13.65 ± 0.29 mg GAE (gallic acid equivalents)/g, while the highest concentration, 96.40 ± 3.44 mg GAE/g, was recorded in the ethanol extract of the leaves. These findings highlight a relatively lower abundance of tannin compounds in the extracts when compared to other phytochemicals.

### 3.3. LC-MS Analysis

Polyphenolic compounds were identified and quantified in both the leaf and root (Table 3 and Table 4) extracts of *C. racemosum*. In the ethanolic leaf extract alone, 14 different phenolic compounds were detected and quantified. The structures of some of the phenolic and flavonoid compounds identified in *C. racemosum* leaf and root extracts are presented in Figure 1.

**Figure 2:**
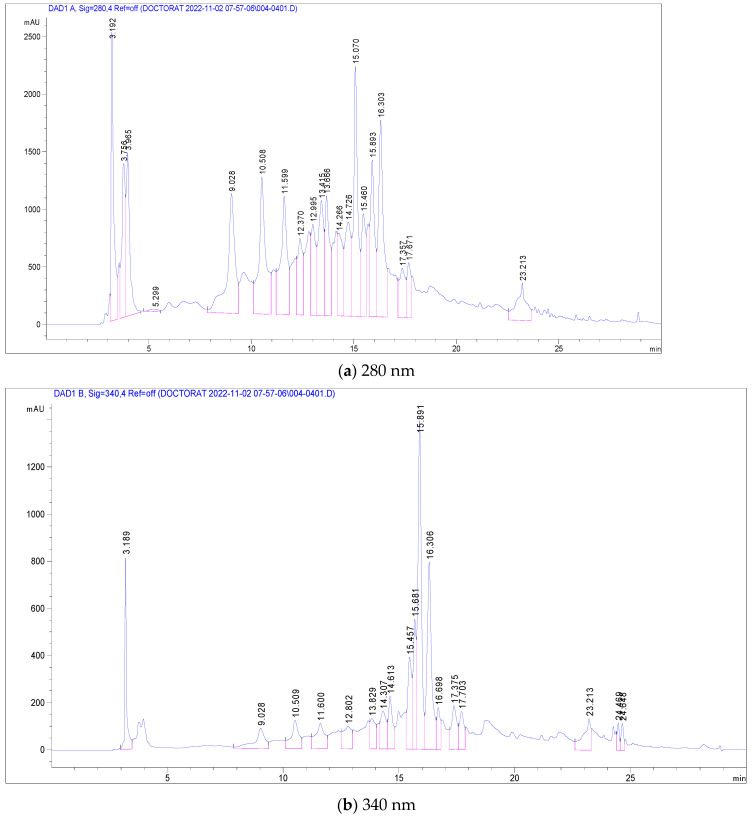
LC-DAD chromatogram of *C. racemosum* leaf extract.

The most abundant among these were Pedunculagin (12,070 mg/g), Vescalagin (10,988 mg/g), Casuarictin (10,958 mg/g), and Digalloyl-glucoside (9226 mg/g). Conversely, compounds found in smaller quantities included Luteolin-galactoside (1085 mg/g), Quercetin-rutinoside (Rutin) at 1752 mg/g, and Ellagic acid-arabinoside (1830 mg/g). The chromatographic analysis, illustrated in Figure 2, displayed peaks at 280 nm and 340 nm, representing these various compounds. This comprehensive analysis provides a detailed profile of the phenolic composition in *C. racemosum*, crucial for understanding its potential health benefits. The maximum absorption of the phenolic compounds was established by examining the UV–Vis spectrum obtained from the DAD records.

In the case of *C. racemosum* root extracts, 14 compounds were identified and quantified in the ethanolic extract, as detailed in Table 4. Notably, Vescalagin, belonging to the Ellagitannin subclass, was present in a significantly high concentration (17,251 mg/mL), surpassing the other compounds. This was followed, in decreasing order of concentration, by Casuarictin, Pedunculagin, Digalloyl-glucoside, Galloyl methyl gallate, 2-Hydroxybenzoic acid, Ellagic acid-glucoside, Punicalin, Galloyl-glucoside, Ellagic acid-arabinoside, Quercetin-glucoside, Luteolin-rhamnoside, Kaempferol-glucoside, and finally Quercetin-rutinoside (Rutin) with the lowest concentration at 0.501 mg/mL.

**Figure 3:**
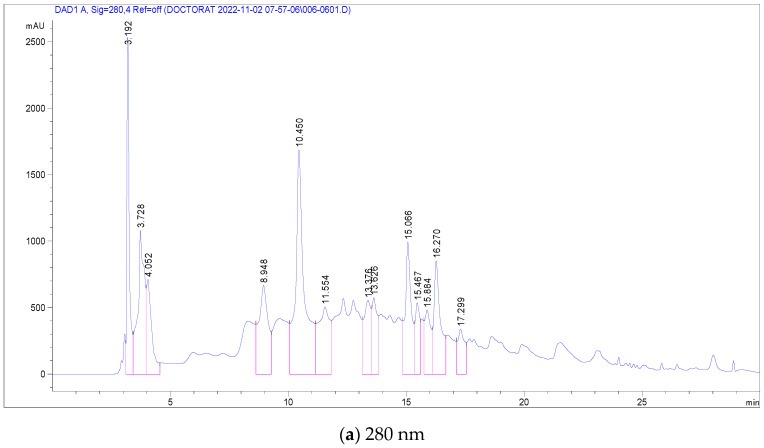

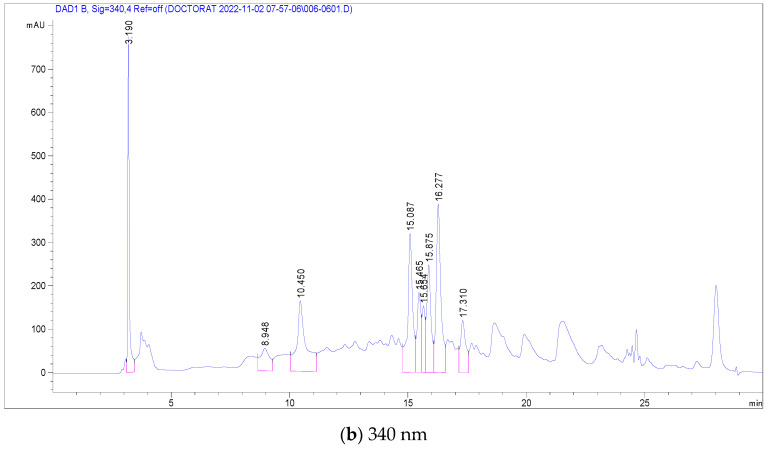
LC-DAD chromatogram of *C. racemosum* root extract.

Figure 3 presents chromatograms that visually display these compounds, marked by distinct peaks. This comprehensive profiling of the phenolic compounds in the root extracts of *C. racemosum* provides valuable insights into the plant’s chemical composition and potential pharmacological properties.

### 3.4. Antimicrobial Activity of C. racemosum Leaf and Root Extracts

#### 3.4.1. Effect of *C. racemosum* Leaf and Root Extracts on Reference Strains

The Minimum Inhibitory Concentrations (MICs) of the aqueous and ethanol extracts of *C. racemosum* varied across different reference strains, as detailed in Table 5. For the leaf extracts, the lowest MIC (0.5 mg/mL) was observed in the ethanolic extract against the *S. aureus* strain, whereas the highest MIC (47.61 mg/mL) was recorded for both the ethanol and aqueous extracts against the *S. enteritidis* strain. In the case of the root extracts, the ethanolic extract again showed the lowest MIC (0.55 mg/mL) against *S. aureus*, but the highest MIC (47.61 mg/mL) was found for the aqueous extract against *L. monocytogenes* and *C. albicans* strains.

Alongside MICs, the Minimum Bactericidal Concentrations (MBCs) were also determined. The lowest MBC for the leaf extracts was 0.55 mg/mL for the ethanol extract against *S. aureus*, followed by the aqueous extract at 2.44 mg/mL. For the root extracts, the lowest MBC (5.14 mg/mL) was observed for the aqueous extract. Although the lowest MIC and MBC values were primarily associated with the *S. aureus* strain, the ratio between these two parameters indicated that the roots’ ethanol extract had a bacteriostatic effect on this strain. A similar bacteriostatic effect was observed for the root ethanol extract on the *S. enteritidis* strain. The other leaf and root extracts demonstrated a bactericidal effect against all the reference strains. This comprehensive analysis of MIC and MBC values provides crucial insights into the antimicrobial potency of the different *C. racemosum* extracts.

#### 3.4.2. Effect of *C. racemosum* Leaf and Root Extracts on Meat Isolated Strains

##### Inhibition Diameters of *C. racemosum* Extracts on Meat Isolated *Staphylococcus* Strains

The interaction between meat-isolated *Staphylococcus* species and *C. racemosum* extracts demonstrated significant variability (*p* < 0.0001). The most substantial inhibitory effect was observed in the acetone root and dichloromethane leaf extracts against the *S. aureus* strain, each yielding an inhibition diameter of 20.00 ± 0.00 mm. Conversely, the smallest inhibition diameter, 9.00 ± 0.00 mm, was noted across three strains: *S. lentus* (for the dichloromethane root extract), *S. cohnii* (for both the water–ethanol leaf and dichloromethane root extracts), and *S. saprophyticus* (for the dichloromethane root extract).

**Figure 4:**
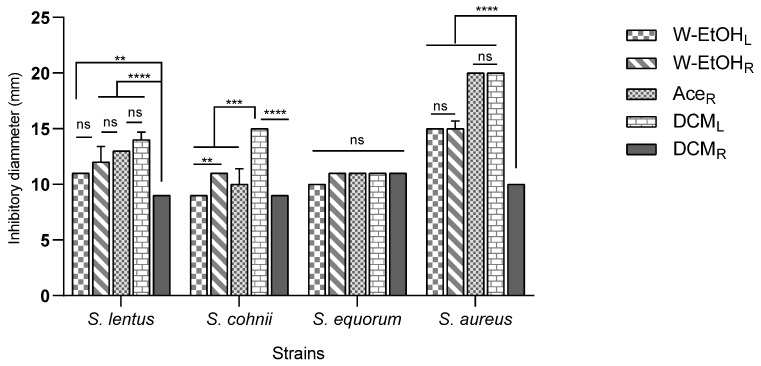

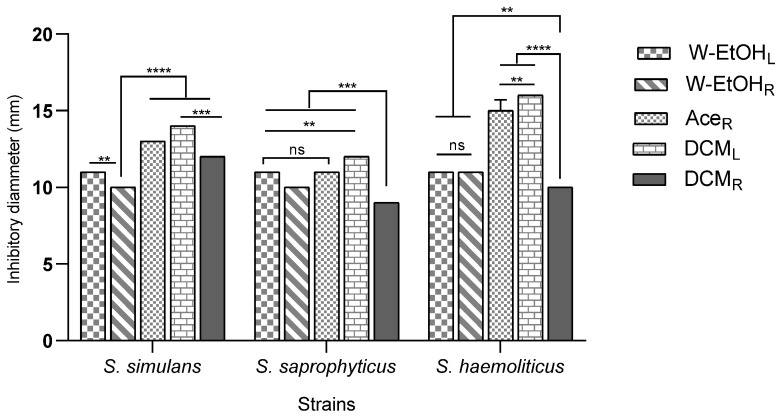
Inhibition diameters of *C. racemosum* extracts on meat-isolated *Staphylococcus* strains in 24h. W-EtOH_L_: water–ethanol leaf extract; DCM_L_: dichloromethane leaf extract; W-EtOH_R_: root water–ethanol extract; DCM_R_: dichloromethane root extract; Ace_R_: acetone root extract; **: *p* < 0.01; ***: *p* < 0.001; ****: *p* < 0.0001; ns: *p* > 0.05.

Additionally, the acetone root and dichloromethane leaf extracts, which exhibited the largest inhibition diameters, also showed considerable efficacy against the *S. haemolyticus* strain. The inhibition diameters for these interactions were 15.00 ± 0.70 mm and 16.00 ± 0.00 mm, respectively, as illustrated in Figure 4. These results underscore the potential of specific *C. racemosum* extracts in exerting strong antimicrobial effects against various *Staphylococcus* species isolated from meat.

##### Minimum Inhibitory Concentration (MIC) and Bactericide/Fungicide (MBC/CMF)

The Minimum Inhibitory Concentration (MIC) and Bactericidal/Fungicidal Concentration (MBC/MFC) of *C. racemosum* extracts displayed variation based on the extract type, plant part, and microbial strain, as detailed in Table 6. For instance, the water–ethanol leaf extract exhibited MICs of 20 mg/mL for all meat-isolated strains, with corresponding MBCs exceeding 20 mg/mL. The dichloromethane leaf extract, however, showed the lowest MIC of 1.25 mg/mL against the *S. haemolyticus* strain. This extract also had MICs of 5 mg/mL for *S. aureus* and *S. lentus*, and 10 mg/mL for *S. simulans* and *S. cohnii*, with MBCs ranging from 5 mg/mL (*S. haemolyticus*) to 20 mg/mL (*S. equorum*).

Similarly, for the root extracts, the water–ethanol extract had an MIC of 20 mg/mL for all strains and MBCs greater than 20 mg/mL. The dichloromethane root extract’s lowest MIC (1.25 mg/mL) was also against *S. haemolyticus*, with MBCs uniformly at 20 mg/mL. Notably, the acetone root extract demonstrated the lowest MICs, varying from 0.156 mg/mL (*S. aureus* and *S. simulans*) to 0.625 mg/mL (*S. equorum*), and MBCs ranging from 1.25 mg/mL (*S. saprophyticus*, *S. aureus*, and *S. simulans*) to 2.5 mg/mL (*S. cohnii*, *S. haemolyticus*, and *S. equorum*).

The analysis of MIC and MBC/MFC ratios revealed that the water–ethanol extracts from both leaves and roots exhibited only bacteriostatic effects on all strains. Conversely, the dichloromethane extracts from leaves and roots exhibited a bactericidal effect on 85.71% of the meat-isolated strains. Despite showing the lowest MIC and MBC, the acetone root extract had a bactericidal effect on 42.85% of these strains. This comprehensive evaluation underscores the varied antimicrobial potential of different *C. racemosum* extracts against specific strains.

The Minimum Inhibitory Concentration (MIC) and Bactericidal/Fungicidal Concentration (MBC/MFC) of *C. racemosum* extracts demonstrate significant variation based on the extract type, plant part, and microbial strain, as detailed in Table 6. For the water–ethanol extracts of the leaves, all MICs against the meat-isolated strains were uniformly 20 mg/mL, with MBCs exceeding this value. In contrast, the dichloromethane leaf extract achieved the lowest MIC of 1.25 mg/mL against *S. haemolyticus*, while also displaying MICs of 5 mg/mL for *S. aureus* and *S. lentus*, and 10 mg/mL for *S. simulans* and *S. cohnii*. The MBCs for this extract varied from 5 mg/mL (*S. haemolyticus*) to 20 mg/mL (*S. equorum).*

Similar patterns were observed in the root extracts, where the water–ethanol extract consistently showed an MIC of 20 mg/mL across all strains, with MBCs exceeding 20 mg/mL. The dichloromethane extract from the roots displayed the lowest MIC of 1.25 mg/mL against *S. haemolyticus* and uniform MBCs of 20 mg/mL. The acetone extract from the roots, however, exhibited the lowest MICs, ranging from 0.156 mg/mL (*S. aureus* and *S. simulans*) to 0.625 mg/mL (*S. equorum*), and MBCs from 1.25 mg/mL (*S. saprophyticus*, *S. aureus*, and *S. simulans*) to 2.5 mg/mL (*S. cohnii*, *S. haemolyticus*, and *S. equorum*).

The analysis of MIC and MBC/MFC ratios revealed that while the water–ethanol extracts from both leaves and roots demonstrated bacteriostatic effects on all strains, the dichloromethane extracts exhibited bactericidal effects on 85.71% of the meat-isolated strains. Despite its lower MIC and MBC, the acetone root extract showed a bactericidal effect on 42.85% of these strains. This detailed analysis underscores the diverse antimicrobial efficacy of different *C. racemosum* extracts against specific microbial strains.

### 3.5. Antioxidant Activity of C. racemosum Leaf and Root Extracts

#### 3.5.1. FRAP

**Figure 5:**
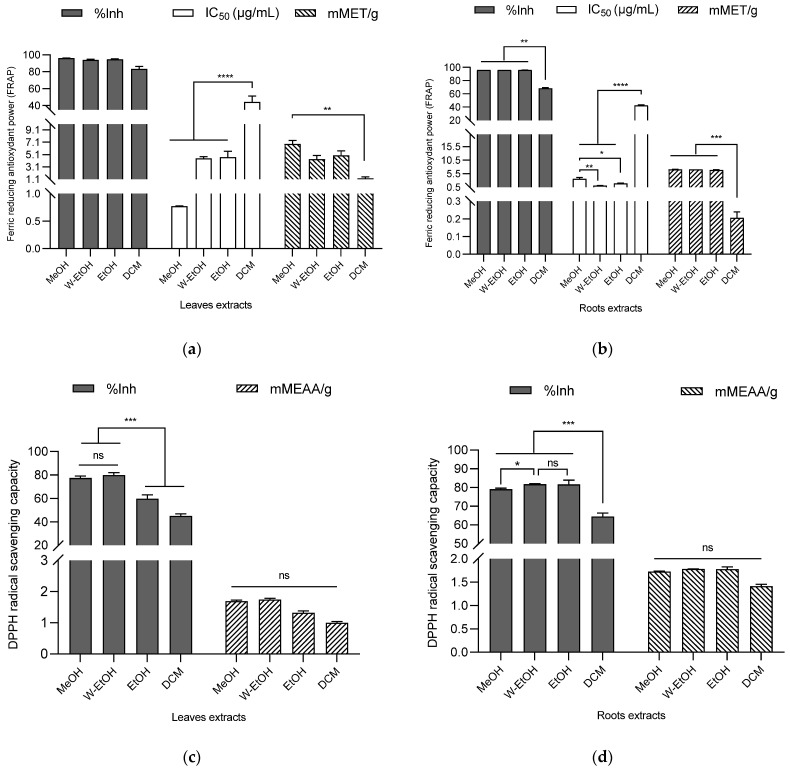
Antioxidant activity of *C. racemosum* leaf and root extracts by FRAP (**a**,**b**) and DPPH (**c**,**d**) methods. W-EtOH: water–ethanol extract; DCM: dichloromethane extract; MeOH: Methanol extract; EtOH: Ethanol extract; *: *p* < 0.05; **: *p* < 0.01; ***: *p* < 0.001; ****: *p* < 0.0001; ns: *p* > 0.05.

The antioxidant potential of the extracts was assessed using DPPH and FRAP assays, with the results indicating a robust antioxidant activity (as shown in Figure 5). In the case of *C. racemosum* root extracts, the percentage of ferric to ferrous ion reduction ranged from 96.17 ± 0.00% in the water–ethanol extract to 68.27 ± 1.10% in the dichloromethane extract. Interestingly, there was no significant difference (*p* > 0.05) in the reduction power between the water–ethanol extract and the methanol (96.15 ± 0.03%) or ethanol extracts (96.04 ± 0.07%).

The water–ethanol extract, which demonstrated the highest reduction percentage, also exhibited the lowest IC_50_ value (1.09 ± 0.09 µg/mL), affirming its potent ferric ion reduction capability. This was followed by the ethanol extract (IC_50_ = 1.90 ± 0.20 µg/mL) and the methanol extract (IC_50_ = 3.60 ± 0.45 µg/mL). In terms of millimolar Trolox equivalents, the water–ethanol extract showed the highest antioxidant content (7.12 ± 0.00 mMET/g), succeeded by the methanol extract (7.08 ± 0.06 mMET/g) and the ethanol extract (6.87 ± 0.14 mMET/g). The dichloromethane extract, in contrast, had the lowest antioxidant content (0.20 ± 0.03 mMET/g). These findings highlight the significant and varying antioxidant capacities of the different *C. racemosum* root extracts.

In the case of *C. racemosum* leaf extracts, the methanol extract exhibited the highest ferric ion reduction percentage (95.98 ± 0.31%), closely followed by the ethanol extract (94.66 ± 0.73%), the water–ethanol extract (94.01 ± 0.71%), and lastly, the dichloromethane extract (83.28 ± 2.99%). There was no significant difference (*p* > 0.05) in the effectiveness of the methanol, ethanol, and water–ethanol extracts, making it challenging to rank them based on efficacy. However, when considering the IC_50_ values, the methanol extract emerged as more potent, with an IC_50_ value three-fold lower (0.77 ± 0.01 µg/mL) than that of the water–ethanol extract (IC_50_ = 4.50 ± 0.26 µg/mL) and the ethanol extract (IC_50_ = 4.66 ± 0.98 µg/mL). In contrast, the dichloromethane extract, with the highest IC_50_ (44.20 ± 6.90 µg/mL), displayed significantly lower activity (*p* < 0.0001) compared to the other extracts.

In terms of Trolox equivalent contents, a decreasing trend was observed: 8.50 ± 0.71 mMET/g for the methanol extract, 6.21 ± 0.91 mMET/g for the ethanol extract, 5.42 ± 0.78 mMET/g for the water–ethanol extract, and 1.55 ± 0.31 mMET/g for the dichloromethane extract. This pattern indicates that among all the leaf extracts, the methanol extract possesses the highest ferric ion reduction activity, highlighting its strong antioxidant potential.

#### 3.5.2. DPPH

Using the DPPH method to assess *C. racemosum* leaf extracts, the water–ethanol extract demonstrated the highest DPPH-radical-scavenging rate at 79.96 ± 2.13%, outperforming the methanol extract (77.39 ± 1.80%), the ethanol extract (59.78 ± 3.24%), and the dichloromethane extract (45.01 ± 1.86%). This trend in scavenging efficiency was further corroborated by the determination of content in millimolar ascorbic acid equivalents (mMEAA/g), as illustrated in Figure 5. In this measurement, the water–ethanol extract again led with the highest content at 1.74 ± 0.04 mMEAA/g, followed closely by the methanol extract (1.68 ± 0.03 mMEAA/g), then the ethanol extract (1.31 ± 0.06 mMEAA/g), and lastly, the dichloromethane extract (0.99 ± 0.03 mMEAA/g).

These results reinforce the observation that the dichloromethane extract had the lowest free DPPH-radical-scavenging activity, consistent with its lower performance in the ferric ion reduction as determined by the FRAP method. This comprehensive analysis highlights the varying antioxidant capabilities of the different *C. racemosum* leaf extracts.

In the case of *C. racemosum* root extracts, a more pronounced antioxidant activity was observed compared to the leaf extracts. This was particularly evident in the results from the dichloromethane extract. When ranking the extracts by their efficiency, the water–ethanol extract maintained its top position with a DPPH-free-radical-scavenging activity of 81.76 ± 0.24%. Notably, the ethanol extract took second place with a scavenging activity of 81.62 ± 2.33%, followed by the methanol extract (79.05 ± 0.62%), and then the dichloromethane extract (64.43 ± 1.89%).

In terms of the millimolar equivalent content of ascorbic acid, the values followed this order: the water–ethanol extract at 1.78 ± 0.00 mMEAA/g, the ethanol extract at 1.77 ± 0.04 mMEAA/g, the methanol extract at 1.72 ± 0.00 mMEAA/g, and the dichloromethane extract at 1.41 ± 0.03 mMEAA/g. Both the DPPH method and the ascorbic acid equivalent content assessment confirmed the potent antioxidant activity of the *C. racemosum* root and leaf extracts, demonstrating their effectiveness in scavenging free radicals.

### 3.6. Anti-Inflammatory Activity of C. racemosum Leaf and Root Extracts

**Figure 6:**
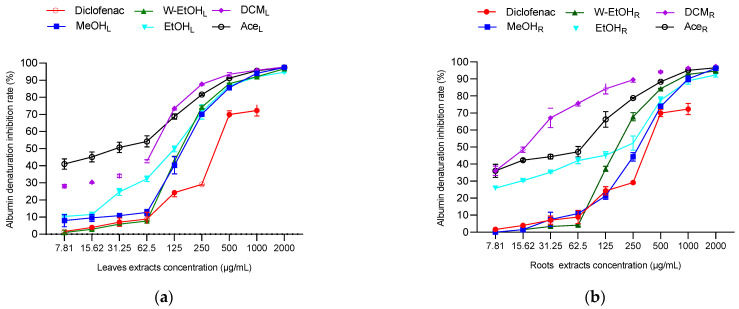

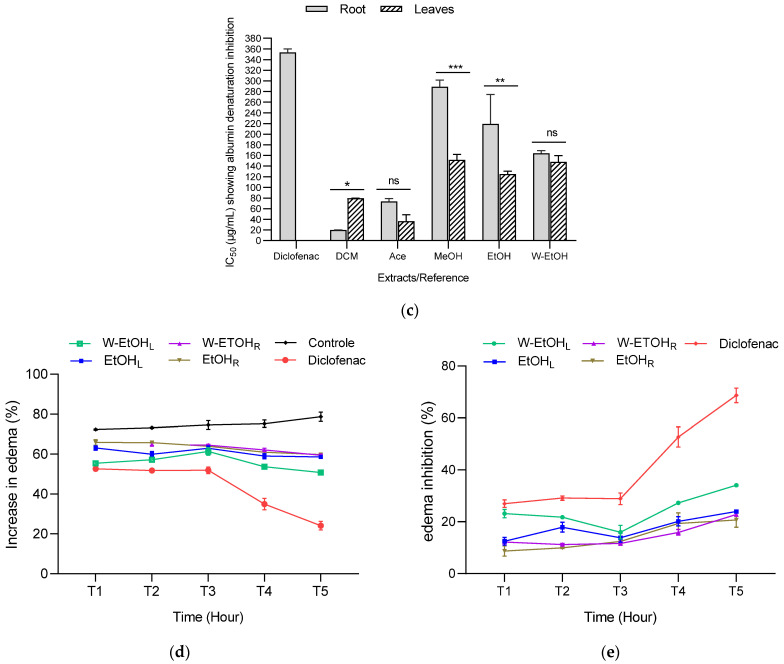
Anti-inflammatory activity of *C. racemosum* leaf and root extracts in in vitro (**a**–**c**) and in vivo (**d**,**e**) models. W-EtOH_L_: water–ethanol leaf extract; EtOH_L_: ethanol leaf extract; W-EtOH_R_: water–ethanol root extract; EtOH_R_: ethanol root extract; MeOH_L_: methanol leaf extract; MeOH_R_: methanol root extract; DCM_L_: dichloromethane leaf extract; DCM_R_: dichloromethane root extract; Ace_L_: acetone leaf extract; Ace_R_: acetone root extract; *: *p* < 0.05; **: *p* < 0.01; ***: *p* < 0.001; ns: *p* > 0.05.

To assess the anti-inflammatory potential of *C. racemosum* leaf and root extracts, both in vitro and in vivo tests were conducted, as illustrated in Figure 6. In the in vitro model, the extracts demonstrated substantial activity at a concentration of 2000 µg/mL, particularly in inhibiting thermal albumin denaturation. This inhibition rate varied depending on the plant part. For instance, the leaf extracts showed inhibition percentages ranging from 97.73 ± 0.05% in the dichloromethane extract to 94.73 ± 0.45% in the ethanol extract. In contrast, for the root extracts, the dichloromethane variant displayed the highest inhibition rate at 97.36 ± 0.05%, while the ethanol extract had the lowest at 92.38 ± 1.26%.

Significant variations were noted in the IC_50_ values between the leaf and root extracts, particularly in the dichloromethane (*p* < 0.05), methanol (*p* = 0.001), and ethanol extracts (*p* = 0.01). The most potent activity was observed in the dichloromethane root extract, which had the lowest IC_50_ value (19.9 ± 0.14 µg/mL), followed by the acetone leaf extract (IC_50_ = 36.6 ± 11.87 µg/mL). Notably, aside from the dichloromethane extract, the methanol and ethanol leaf extracts exhibited lower IC_50_ values (151.6 ± 10.46 µg/mL and 125.05 ± 5.16 µg/mL, respectively) compared to their root counterparts (Figure 6c). When compared to diclofenac, a reference anti-inflammatory molecule, the extracts showed higher efficacy in inhibiting thermal albumin denaturation, indicating their strong anti-inflammatory properties.

The anti-inflammatory activity of *C. racemosum* leaf and root extracts was further evaluated using a model of acute edema in rat hind paws, induced by a 1% carrageenan solution, as depicted in Figure 6d,e. The edema size was measured at one-hour intervals over a five-hour period, and the percentages of edema increase and inhibition were calculated. The results are presented in Figure 6.

In control rats, 1% carrageenan induced edema in a proportion that ranged from 72.33 ± 0.29% (after 1 h) to 78.73 ± 2.29% (after 5 h), confirming the effectiveness of the method in inducing edema. Additionally, the administration of the extracts at a dosage of 200 mg/kg body weight, and diclofenac at 50 mg/kg, successfully prevented edema in the treated rats compared to the control rats, which only received physiological water. The level of prevention varied with the different extracts and over time.

For most extracts, the increase in edema decreased progressively from the first hour to the fifth hour (Figure 6d). For instance, with the hydroethanolic leaf extract, the increase in edema decreased from 55.42 ± 1.16% (after 1 h) to 50.82 ± 0.24% (after 5 h). In the case of the ethanol leaf extract, the increase in edema reduced from 63.12 ± 1.07% (after 1 h) to 58.61 ± 0.28% (after 5 h). Similarly, for the root extracts, the hydroethanolic extract showed a decrease in edema from 63.35 ± 0.11% (after 1 h) to 59.49 ± 0.59% (after 5 h), while the ethanol extract exhibited a reduction from 65.84 ± 1.41% (after 1 h) to 59.79 ± 0.20% (after 5 h). These results demonstrate the varying degrees of anti-inflammatory efficacy of *C. racemosum* extracts in a time-dependent manner.

The observed inhibition of edema increase indicates that diclofenac effectively prevented edema formation, achieving up to a 68.69 ± 2.79% reduction, as shown in Figure 6e. When examining the extracts, the water–ethanol extract of the leaves exhibited the highest inhibition rate at 34.30 ± 0.31% after five hours (T5). This was followed by the ethanol leaf extract (23.99 ± 0.36%), the hydroethanolic root extract (22.84 ± 0.77%), and finally the ethanol root extract (20.66 ± 2.78%). In comparison to diclofenac, these extracts displayed moderate anti-inflammatory activity, suggesting their potential as alternative treatments, albeit with a lesser degree of efficacy than the standard anti-inflammatory drug diclofenac.

### 3.7. Human Melanoma Cells (A375) Proliferation Assay

**Figure 7:**
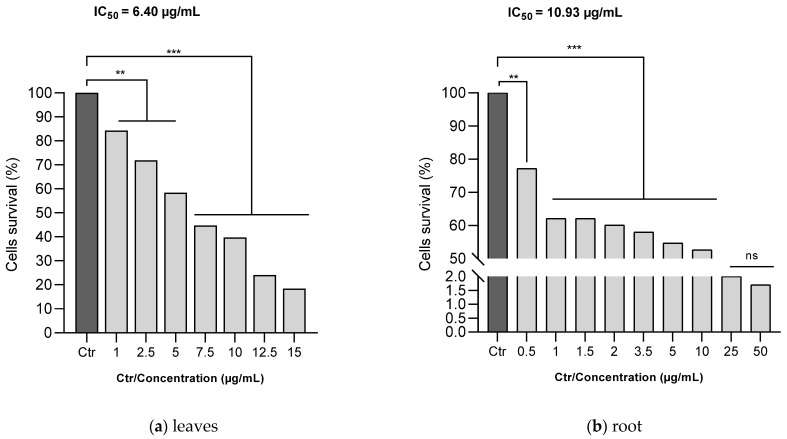
Effects of ethanol *C. racemosum* leaf and root extracts on human melanoma cells (A375) cell viability. **: *p* < 0.01; ***: *p* < 0.001; ns: *p* > 0.05.

The MTT assay was utilized to evaluate the antiproliferative efficacy of ethanol *C. racemosum* leaf and root extracts on human melanoma cells (A375). As depicted in Figure 7, both the leaf and root extracts of *C. racemosum* reduced cell viability in a dose-dependent manner. At a concentration of 1 µg/mL, the leaf extracts significantly reduced (*p* < 0.01) cell viability from 100% (control) to 84.3%, whereas the root extracts decreased it more substantially to 62.1% (*p* < 0.001). This indicates that at this concentration, the root extracts were notably more effective in inhibiting cell proliferation compared to the leaf extracts.

Furthermore, it was observed that the leaf extract inhibited the proliferation of 50% of the cells at a concentration as low as 7.5 µg/mL. In contrast, a similar level of inhibition in the roots was observed within a concentration range of 10 to 25 µg/mL. The IC_50_ values corroborated these findings, showing that the leaf extract had a more pronounced antiproliferative activity than the root extract, with the IC_50_ decreasing from 10.93 µg/mL for the root extract to 6.40 µg/mL for the leaf extract.

Despite these differences, it is important to note that both the leaf and root extracts exhibited effective antiproliferative actions against human melanoma cells (A375) even at low doses, underscoring their potential as agents in cancer therapy.

## 4. Discussion

*C. racemosum*, a member of the Combretaceae family, is recognized in the traditional Beninese pharmacopoeia and utilized by local populations for various medicinal purposes. Despite its widespread use, there is a notable lack of scientific data on samples from Benin. This study aimed to identify bioactive compounds in *C. racemosum*’s leaves and roots to assess their biological activities. Preliminary screenings revealed the presence of several secondary metabolites such as alkaloids, tannins, flavonoids, quinone derivatives, and free anthracenics in both the leaves and roots. Previous research [15,38,39,40] has similarly identified these compounds in the leaves, underscoring their broad range of biological activities [41,42,43,44].

These compounds were extracted using ultrasonication, a method known for enhancing solvent penetration, reducing extraction time, and increasing yield by causing fragmentation, sonocapillary effects, and sonoporation [45]. Subsequent analysis quantified the total polyphenols, flavonoids, and hydrolyzable and condensed tannins in various proportions in the extracts. The results indicated that both the leaves and roots were richer in total polyphenols and flavonoids compared to tannins, with similar polyphenol concentrations in roots (5347.19 ± 99.76 mgGAE/100 g) and leaves (5316.98 ± 99.15 mgGAE/100 g). However, flavonoids were more abundant in leaves (13,157.10 ± 28.43 mgQE/100 g) than in roots (10,888.12 ± 375.10 mgQE/100 g).

Flavonoids, widely abundant in plants [46,47], vary in quantity based on factors like plant genotype, environmental conditions, and physical stresses [48]. Quantified as Quercetin equivalents in this study, flavonols like Quercetin and Kaempferol are notably abundant in green leaves [49,50]. The high flavonoid content in leaves could be attributed to their exposure to light and temperature, factors that influence flavonoid biosynthesis. This study’s findings align with the hypothesis that environmental exposure can impact flavonoid levels in plant leaves.

A total of 14 phenolic compounds were successfully identified and quantified in the ethanolic extracts of *C. racemosum* leaves and roots through LC-MS analysis. In the leaf extract, the most prevalent compounds were identified as Pedunculagin (12,070 mg/g), Vescalagin (10,988 mg/g), Casuarictin (10,958 mg/g), and Digalloyl-glucoside (9226 mg/g). Conversely, in the root extract, Vescalagin, particularly from the Ellagitannin subclass, stood out with a significantly higher concentration (17,251 mg/mL) compared to other compounds. These identified compounds are notable for their varied biological activities, highlighting the therapeutic potential of *C. racemosum*’s extracts.

This study’s investigation into antimicrobial activity revealed that the active extracts from *C. racemosum* exhibited a bactericidal effect on the tested strains, with the notable exceptions of *S. aureus* and *S. enteridis*. This remarkable antimicrobial activity is likely attributable to the compounds identified in the plant’s leaves and roots. Specifically, the efficacy of hydrolyzable tannins such as Punicalin, a member of the Ellagitannin subclass present in *C. racemosum*, has been documented by various researchers ([51,52]). Tannins, as Daglia [53] notes, are categorized into two subclasses: proanthocyanidins (condensed tannins) and gallotannins and ellagitannins (hydrolyzable tannins). Our LC-MS analysis revealed the presence of numerous gallotannins and ellagitannins.

Puupponen-Pimia et al. [54] have reported that ellagitannins are particularly notable for their antimicrobial properties, as they can inhibit the growth of certain Gram-negative intestinal bacteria to varying degrees. Gallotannins are known for their strong affinity for iron and their capability to inactivate membrane-bound proteins. The antimicrobial mechanisms of tannins are diverse, and their mode of action is likely species-specific [55]. One proposed mechanism for the antimicrobial action of tannins is the inactivation of microbial adhesins and cell envelope transport proteins [56], which could explain the broad spectrum of antimicrobial activity observed in this study.

Besides their antimicrobial properties, the leaf and root extracts of *C. racemosum* also exhibited notable antioxidant and anti-inflammatory activities. The antioxidant activity was pronounced, while the anti-inflammatory effect varied, showing moderate efficacy in the edema model but strong activity in protein denaturation inhibition. These activities are likely related to the composition of the extracts, as revealed by LC-MS analysis, which identified a variety of flavonoid compounds. Flavonoids are renowned for their excellent antioxidant properties, functioning through mechanisms such as scavenging free radicals and reactive oxygen species (ROS), chelating metals, and preventing the oxidation of low-density lipoproteins (LDLs) [57].

The antioxidant capacity of the leaf and root extracts was assessed using DPPH and FRAP methods, where flavonoids were found in significant quantities and identified through LC-MS. These flavonoids, particularly Luteolin and Quercetin, present in both the leaf and root extracts, are believed to contribute to the observed antioxidant and anti-inflammatory activities. Luteolin, a flavone metabolite, is known for its antioxidant properties, attributed to its C6-C3-C6 structure with multiple hydroxyl groups [58]. It also plays a role in activating and resolving inflammation pathways [59].

The study noted that the extracts showed greater anti-inflammatory activity in inhibiting protein denaturation compared to edema inhibition. This difference could be due to the interaction mechanisms of the compounds in the extracts, especially phenolic compounds, with proteins. Protein–phenolic interactions can occur via covalent and non-covalent mechanisms, influenced by various factors like protein characteristics, phytochemical types, protein/phytochemical ratios, temperature, pH, ionic flux, and more [60,61].

The in vitro and in vivo models used in this study could account for the observed differences. In vivo parameters such as the rats’ diet, physiology, metabolism, and internal pH can affect the activity of the extracts, possibly explaining the moderate anti-inflammatory activity seen in vivo. Research by Seczyk et al. [62] on interactions between standard phenolic compounds and protein fractions like albumins supports this, showing that the type of phenolic significantly affects protein–phenolic interactions. This comprehensive analysis suggests that the bioactive compounds in *C. racemosum*, particularly flavonoids and phenolics, are key contributors to its antioxidant and anti-inflammatory properties.

The antiproliferative potential of *C. racemosum* leaf and root extracts was investigated using a human melanoma cell model. Melanoma, a malignant form of skin cancer originating from melanocytes, is one of the most aggressive and deadly cancers, known for its rapid increase in incidence as per World Health Organization estimates [63]. It has particularly low survival rates [64], underscoring the urgent need for effective treatments.

Given the high treatment costs in developed countries and the challenges in healthcare accessibility in regions like Benin, exploring natural compounds for treating melanoma is vital. This study demonstrated that *C. racemosum* leaf and root extracts, sourced from Benin, significantly (*p* < 0.001) inhibited the proliferation of human melanoma cells (A375), even at low concentrations. This aligns with other research showcasing the antiproliferative effects of various plant extracts on melanoma cells, including *Salvia fructicosa* [65], *Polypodium vulgare* [66], and selected Saudi plants [67]. Many of the mechanisms of cell death have overlapping signaling pathways, which can be a challenge with treatment and emerging resistance. Various factors affect the fate of a cell to undergo apoptosis, autophagy, or necroptosis [68], including energy/ATP levels, the degree of damage or stress, and the presence of specific pathway inhibitors [69]. Some studies like [68] are successfully investigating the anticancer activity of pangolin scale extract using melanoma A375 cell lines. These authors show that pangolin scale extract inhibits the proliferation and migration of melanoma cells and induces apoptosis. Whole-transcriptome analysis performed by the authors of [68] reveals that PSE may cause cell cycle arrest in melanoma cells and promote apoptosis, mainly by upregulating the p53 signaling pathway and downregulating the PI3K-Akt signaling pathway. With the mechanism of certain natural products such as PSE in the inhibition of the proliferation of A375 melanoma cells being known, there remains a prospect in our future work of elucidating the mechanism of *C. racemosum* extracts in the proliferation inhibition of A375 melanoma cells.

The findings of this study lay foundational groundwork for further anticancer investigations into *C. racemosum* extracts, highlighting their potential as a natural therapeutic option against melanoma. This is especially relevant for regions where conventional treatments are less accessible, offering a promising avenue for affordable and effective cancer management.

## 5. Conclusions

*C. racemosum* leaf and root extracts were found to contain a range of polyphenolic compounds, including Pedunculagin, Vescalagin, Casuarictin, Digalloyl-glucoside, Luteolin-galactoside, Quercetin-rutinoside (Rutin), Ellagic acid-arabinoside, Kaempferol-glucoside, 2-Hydroxybenzoic acid, Punicalin, and Galloyl methyl gallate. These compounds are known for their significant biological activities, encompassing antimicrobial, antioxidant, anti-inflammatory, and antiproliferative effects.

Both the leaf and root extracts exhibited bactericidal properties against various reference and meat-isolated strains. In terms of antioxidant activity, the dichloromethane extract displayed the lowest DPPH-free-radical-scavenging activity, a finding corroborated by its reduced ferric ion reduction as per the FRAP method. Nonetheless, both the FRAP and DPPH assays consistently demonstrated the potent antioxidant capabilities of *C. racemosum* leaf and root extracts.

When assessing anti-inflammatory properties, the extracts showed a greater ability to inhibit protein denaturation compared to reducing edema. Notably, the leaf extracts manifested more pronounced antiproliferative effects than the root extracts, as evidenced by lower IC_50_ values, decreasing from 10.93 µg/mL (root extract) to 6.40 µg/mL (leaf extract). These findings lay a solid foundation for future research into the anticancer potential of *C. racemosum* extracts, suggesting their possible application in natural cancer therapy strategies.

## Figures and Tables

**Table 1:** Phytochemical screening of *C. racemosum* organs (leaves and roots).

Secondary Metabolites	*C. racemosum* Leaves	*C. racemosum* Roots
Catechic tannins	+	+
Gallic tannins	+	+
Flavonoids	+	+
Anthocyanins	+	+
Saponosides	−	+
Alkaloids	+	+
Quinone derivatives	+	+
Leuco-anthocyanins	−	−
Cyanogenic derivatives	−	−
Mucilages	+	−
Reducing compounds	+	−
Free anthracenics	+	+
O. Heterosides	−	−
O. glycosides with reduced genins	−	−

(+): Presence of secondary metabolite. (−): Absence of secondary metabolite.

**Table 2:** Polyphenol, flavonoid, and tannin contents of *C. racemosum* extracts.

Part	Extracts	Polyphenols (mgEGA/100 g)	Flavonoids (mgEQ/100 g)	Condensed Tannins (mgEC/g)	Hydrolysable Tannins (mgEGA/g)
Leaves	Water	4953.62 ± 918.10	7562.03 ± 745.01	nd	nd
	Ethanol	5316.98 ± 99.15	13,157.10 ± 28.43	35.36 ± 4.71	96.40 ± 3.44
	Water–ethanol	1749.78 ± 301.34	1132.65 ± 89.70	35.23 ± 3.05	88.95 ± 4.99
	Dichloromethane	413.20 ± 12.56	2168.50 ± 12.10	25.48 ± 0.56	13.65 ± 0.29
	Methanol	1585.28 ± 157.67	979.55 ± 53.62	29.85 ± 2.77	91.11 ± 2.32
	Acetone	1548.48 ± 226.49	756.68 ± 76.41	39.95 ± 1.42	77.05 ± 1.70
Root	Water	4483.90 ± 975.23	3221.36 ± 450.37	-	-
	Ethanol	5347.19 ± 99.76	10,888.12 ± 375.10	24.65 ± 2.99	85.09 ± 19.23
	Water–ethanol	1608.01 ± 219.20	961.14 ± 96.16	22.92 ± 3.66	70.91 ± 4.33
	Dichloromethane	1666.37 ± 148.03	2616.14 ± 197.42	396.75 ± 12.76	52.48 ± 2.04
	Methanol	1706.49 ± 68.72	920.44 ± 64.50	24.13 ± 2.89	88.49 ± 7.36
	Acetone	4654.54 ± 27.74	1466.95 ± 21.02	33.62 ± 0.76	22.48 ± 0.63

nd: not determined.

**Table 3:** LC-DAD and MS data obtained after positive ionization of ethanolic *C. racemosum* leaf extracts and the content of phenolic compounds, expressed in mg/g.

Peak No.	R_t_ (min)	UV λ_max_ (nm)	[M + H]^+^ (*m*/*z*)	Phenolic Compound	Subclass	mg/g
1	3.21	270	139	2-Hydroxybenzoic acid	Hydroxybenzoic acid	6.457
2	3.76	278	337	Galloyl methyl gallate	Gallic derivative	4.465
3	3.98	276	333	Galloyl-glucoside	Gallic derivative	5.185
4	9.03	260, 360	935	Casuarictin	Ellagitannin	10.958
5	10.51	260, 360	935	Vescalagin	Ellagitannin	10.988
6	11.59	277	485	Digalloyl-glucoside	Gallotannin	9.226
7	13.41	260	465, 303	Ellagic acid-glucoside	Hydroxybenzoic acid	7.352
8	13.67	280	783	Punicalin	Ellagitannin	6.248
9	14.61	350, 260	449, 287	Luteolin-galactoside	Flavone	1.085
10	15.02	280	785	Pedunculagin	Ellagitannin	12.070
11	15.45	260	435, 303	Ellagic acid-arabinoside	Hydroxybenzoic acid	1.830
12	15.68	360, 260	611, 303	Quercetin-rutinoside (Rutin)	Flavonol	1.752
13	15.89	350, 260	433, 287	Luteolin-rhamnoside	Flavone	5.547
14	16.31	360, 260	465, 303	Quercetin-glucoside	Flavonol	4.005
				**Total phenolics**		**87.168**

**Table 4:** LC-DAD and MS data obtained after positive ionization of ethanolic *C. racemosum* root extracts and the content of phenolic compounds, expressed in mg/g.

Peak No.	R_t_ (min)	UV λ_max_ (nm)	[M+H]^+^ (*m*/*z*)	Phenolic Compound	Subclass	mg/g
1	3.19	270	139	2-Hydroxybenzoic acid	Hydroxybenzoic acid	5.378
2	3.72	278	337	Galloyl methyl gallate	Gallic deriv.	6.127
3	4.05	276	333	Galloyl-glucoside	Gallic deriv.	3.292
4	8.95	260, 360	935	Casuarictin	Ellagitannin	7.225
5	10.45	260, 360	935	Vescalagin	Ellagitannin	17.251
6	11.55	277	485	Digalloyl-glucoside	Gallotannin	6.926
7	13.41	260	465, 303	Ellagic acid-glucoside	Hydroxybenzoic acid	4.420
8	13.67	280	783	Punicalin	Ellagitannin	3.796
9	15.06	280	785	Pedunculagin	Ellagitannin	7.022
10	15.46	260	435, 303	Ellagic acid-arabinoside	Hydroxybenzoic acid	3.019
11	15.65	360, 260	611, 303	Quercetin-rutinoside (Rutin)	Flavonol	0.501
12	15.87	350, 260	433, 287	Luteolin-rhamnoside	Flavone	1.165
13	16.27	360, 260	465, 303	Quercetin-glucoside	Flavonol	1.978
14	17.31	350, 250	449, 287	Kaempferol-glucoside	Flavonol	0.717
				**Total phenolics**		**68.817**

**Table 5:** Minimum Inhibitory (MIC) and Bactericidal/Fungicidal Concentration (MBC/MFC) of *C. racemosum* leaf and root extracts.

	Extracts	Parameters (mg/mL)	*S. aureus*	*E. coli*	*S. enteritidis*	*L. monocytogenes*	*C. albicans*
Leaves	Water	MIC	1.1	22.67	47.61	22.67	22.67
		MBC	2.44	22.67	47.61	47.61	47.61
		MBC/MIC	2.21 *	1 *	1 *	2.10 *	2.10 *
	Ethanol	MIC	0.5	16.73	47.61	22.67	22.67
		MBC	0.55	47.61	47.61	22.67	22.67
		MBC/MIC	1.1 *	2.84 *	1 *	1 *	1 *
Roots	Water	MIC	2.44	22.67	5.14	47.61	47.61
		MBC	5.14	47.61	10.79	47.61	47.61
		MBC/MIC	2.10 *	2.10 *	2.09 *	1 *	1 *
	Ethanol	MIC	0.55	5.14	5.14	10.79	10.79
		MBC	22.67	10.79	22.67	22.67	22.67
		MBC/MIC	41.21	2.09 *	4.41	2.10 *	2.10 *

The ratio MBC/MIC value with * = Bactericidal effects and without * = Bacteriostatical effects.

**Table 6:** Minimum Inhibitory Concentration (MIC) and Bactericide/Fungicide (CMB/CMF) of the different extracts on meat isolated Staphylococcus strains.

	Extracts	Parameters (mg/mL)	*S. sap*	*S. aur*	*S. len*	*S. sim*	*S. coh*	*S. hae*	*S. equ*
Leaves	W-EtOH	MIC	20	20	20	20	20	20	20
		MBC	>20	>20	>20	>20	>20	>20	>20
		MBC/MIC	nd	nd	nd	nd	nd	nd	nd
	DCM	MIC	10	5	5	10	10	1.25	20
		MBC	20	20	20	20	20	5	>20
		MBC/MIC	2 *	4	4	2 *	2 *	4	nd
Roots	W-EtOH	MIC	20	20	20	20	20	20	20
		MBC	>20	>20	>20	>20	>20	>20	>20
		MBC/MIC	nd	nd	nd	nd	nd	nd	nd
	DCM	MIC	10	10	10	5	5	1.25	5
		MBC	20	20	20	20	20	20	20
		MBC/MIC	2 *	2 *	2 *	4	4	16	4
	Ace	MIC	0.312	0.156	1.25	0.156	1.25	0.312	0.625
		MBC	1.25	1.25	5	1.25	2.5	2.5	2.5
		MBC/MIC	4.01	8.01	4	8.01	2 *	8.01	4

*S. sim*: *Staphylococcus simulans*; *S. len*: *Staphylococcus lentus*; *S. aur*: *Staphylococcus aureus*; *S. equ*: *Staphylococcus equorum*; *S. hae*: *Staphylococcus haemolyticus*; *S. coh*: *Staphylococcus cohnii*; *S. sap*: *Staphylococcus saprophyticus*. The MBC/MIC ratio: * = bactericidal effects and without; * = bacteriostatic effects.

## Data Availability

All data is contained within the article.
